# Evaluating the Impact of Applying Personal Glucose Targets in a Closed-Loop System for People With Type 1 Diabetes

**DOI:** 10.1177/19322968221145184

**Published:** 2022-12-20

**Authors:** Mustafa Fattah, Charlotte K. Boughton, Julia Ware, Janet M. Allen, Sara Hartnell, Malgorzata E. Willinska, Ajay Thankamony, Carine de Beaufort, Fiona M. Campbell, Elke Fröhlich-Reiterer, Sabine E. Hofer, Thomas M. Kapellen, Birgit Rami-Merhar, Atrayee Ghatak, Tabitha L. Randell, Rachel E. J. Besser, Daniela Elleri, Nicola Trevelyan, Louise Denvir MD, Nikki Davis, Lia Bally, Hood Thabit, Lalantha Leelarathna, Mark L. Evans, Julia K. Mader, Roman Hovorka

**Affiliations:** 1 Wellcome-MRC Institute of Metabolic Science, University of Cambridge, Cambridge, UK; 2 Wolfson Diabetes and Endocrine Clinic, Cambridge University Hospitals NHS Foundation Trust, Cambridge, UK; 3 University of Cambridge Metabolic Research Laboratories and NIHR Cambridge Biomedical Research Centre, Wellcome Trust-MRC Institute of Metabolic Science, Addenbrooke’s Hospital, Cambridge, UK; 4 Department of Paediatrics, University of Cambridge, Cambridge, UK; 5 Diabetes & Endocrine Care Clinique Pediatrique, Pediatric Clinic/Centre Hospitalier de Luxembourg, Luxembourg, Luxembourg; 6 Faculty of Science, Technology and Medicine, University of Luxembourg, Esch-Belval, Luxembourg; 7 Department of Paediatric Diabetes, Leeds Children’s Hospital, Leeds, UK; 8 Department of Pediatrics, Medical University of Graz, Graz, Austria; 9 Department of Pediatrics, Medical University of Innsbruck, Innsbruck, Austria; 10 Hospital for Children and Adolescents, University of Leipzig, Leipzig, Germany; 11 Department of Pediatrics and Adolescent Medicine, Medical University of Vienna, Vienna, Austria; 12 Alder Hey Children’s NHS Foundation Trust, Liverpool, UK; 13 Nottingham Children’s Hospital, Nottingham, UK; 14 Department of Paediatrics, University of Oxford, Oxford, UK; 15 NIHR Oxford Biomedical Research Centre, John Radcliffe Hospital, Oxford, UK; 16 Royal Hospital for Children & Young People, Edinburgh, UK; 17 Southampton Children’s Hospital, Southampton, UK; 18 Department of Diabetes, Endocrinology, Nutritional Medicine and Metabolism, Inselspital, Bern University Hospital and University of Bern, Bern, Switzerland; 19 Diabetes, Endocrinology and Metabolism Centre, Manchester University NHS Foundation Trust, Manchester, UK; 20 Division of Diabetes, Endocrinology and Gastroenterology, Faculty of Biology, Medicine and Health, University of Manchester, Manchester, UK; 21 Division of Endocrinology and Diabetology, Department of Internal Medicine, Medical University of Graz, Graz, Austria

**Keywords:** algorithm, glucose control, hybrid closed-loop, personal glucose target, type 1 diabetes

## Abstract

**Background:**

CamAPS FX is a hybrid closed-loop smartphone app used to manage type one diabetes. The closed-loop algorithm has a default target glucose of 5.8 mmol/L (104.5 mg/dL), but users can select personal glucose targets (adjustable between 4.4 mmol/L and 11.0 mmol/L [79 mg/dL and 198 mg/dL, respectively]).

**Method:**

In this post-hoc analysis, we evaluated the impact of personal glucose targets on glycemic control using data from participants in five randomized controlled trials.

**Results:**

Personal glucose targets were widely used, with 20.3% of all days in the data set having a target outside the default target bin (5.5-6.0 mmol/L [99-108 mg/dL]). Personal glucose targets >6.5 mmol/L (117 mg/dL) were associated with significantly less time in target range (3.9-10.0 mmol/L [70-180 mg/dL]; 6.5-7.0 mmol/L [117-126 mg/dL]: mean difference = −3.2 percentage points [95% CI: −5.3 to −1.2; *P* < .001]; 7.0-7.5 mmol/L [126-135 mg/dL]: −10.8 percentage points [95% CI: −14.1 to −7.6; *P* < .001]). Personal targets >6.5 mmol/L (117 mg/dL) were associated with significantly lower time (<3.9 mmol/L [<70 mg/dL]; 6.5-7.0 mmol/L [117-126 mg/dL]: −1.85 percentage points [95% CI: −2.37 to −1.34; *P* < .001]; 7.0-7.5 mmol/L [126-135 mg/dL]: −2.68 percentage points [95% CI: −3.49 to −1.86; *P* < .001]).

**Conclusions:**

Discrete study populations showed differences in glucose control when applying similar personal targets.

## Introduction

CamAPS FX (CamDiab, Cambridge, UK) is an interoperable hybrid closed-loop smartphone app used to manage type one diabetes (T1D). The app receives sensor glucose data from the Dexcom G6 CGM system (Dexcom, San Diego, CA, USA), and the algorithm adjusts insulin administration via a compatible insulin pump (Dana RS or Dana i; Sooil, Seoul, South Korea, or Ypsopump; Ypsomed, Burgdorf, Switzerland). The closed-loop algorithm has a default target glucose level of 5.8 mmol/L (104.5 mg/dL), but users can select personal glucose targets, adjustable between 4.4 mmol/L (79 mg/dL) and 11.0 mmol/L (198 mg/dL) in 0.1 mmol/L (1.8 mg/dL) increments, which can be set differently for each half-hour block of the 24-hour period.

In this post-hoc analysis, we aimed to evaluate the impact of user-selected personal glucose targets on glycemic control.

## Methods

We analyzed data from participants with T1D using CamAPS FX in five randomized controlled trials across different demographic cohorts: very young children aged one to seven years,^1^ children and adolescents aged six to 19 years,^2^ adolescents aged 10 to 17 years using closed-loop from diagnosis,^3^ adults aged ≥18 years,^4^ and older adults aged ≥60 years.^5^ All studies received regulatory and ethical approval; participants/guardians signed informed consent. Selection of personal targets was determined by the user and could be adjusted throughout the study to suit the needs of the user.

We included 18 484 days of data from 185 participants for whom the closed-loop system was enabled for at least 70% of the 24-hour period. Data of each day were binned into 0.5 mmol/L (9.0 mg/dL) bins (4.0-11.0 mmol/L [72-198 mg/dL]) according to the average of the personal glucose targets applied that day. Only bins with a minimum of 14 days of data were included in the analysis. Data from each target bin were compared using analysis of variance with the post-hoc Tukey test for pairwise comparisons with the default target bin (5.5-6.0 mmol/L [99-108 mg/dL]). Statistical analyses were performed using SPSS (Version 27.0; IBM Corp., Armonk, NY, USA). *P* values <.05 were considered statistically significant.

## Results

Personal glucose targets were widely used, with 20.3% of all days in the data set having a personal target outside the default target bin (5.5-6.0 mmol/L [99-108 mg/dL]). Over 95% of customized personal targets were set above the default target. Personal targets were used most frequently for very young children (>25% of days) and then older adults (>20% of days), while older children and adolescents used this functionality the least (<10% of days).

**Figure 1.**
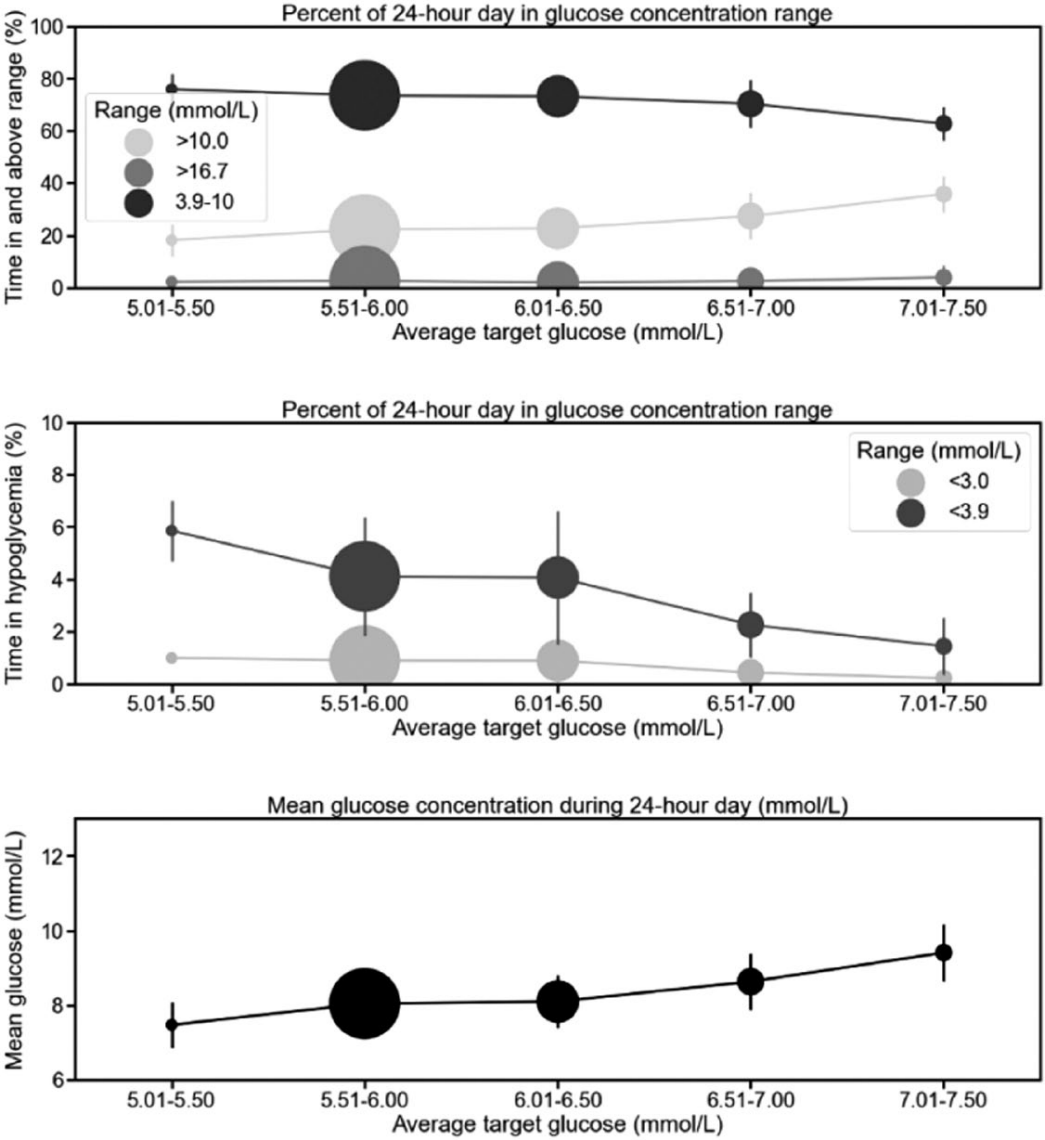
Glycemic metrics across different personal glucose target bins. The circle size reflects the *n* number with average personal glucose targets within the bin.

The mean glucose level increased significantly with a higher personal target (6.5-7.0 mmol/L [117-126 mg/dL] bin: mean difference 0.59 mmol/L [10.6 mg/dL; 95% CI: 0.41-0.77 mmol/L, 7-14 mg/dL; *P* < .001]; 7.0-7.5 mmol/L [126-135 mg/dL] bin: 1.36 mmol/L [24.5 mg/dL; 95% CI: 1.08-1.65 mmol/L, 19-30 mg/dL; *P* < .001]) and was significantly lower with a lower personal target (5.0-5.5 mmol/L [90-99 mg/dL] bin: mean difference −0.57 mmol/L [10.3 mg/dL; −1.00 to −0.14 mmol/L, −18 to −3 mg/dL; *P* < .01]) than when the default target was applied (Table 1 and Figure 1).

**Table 1.** Glycemic Metrics by Personal Glucose Target Bin.

	Mean glucose (mmol/L) [mg/dL]	Time in sensor glucose range (%)				
		3.9-10.0 mmol/L (70-180 mg/dL)	<3.9 mmol/L (70 mg/dL)	<3.0 mmol/L (54 mg/dL)	>10.0 mmol/L (180 mg/dL)	>16.7 mmol/L (301 mg/dL)
Personal glucose target bin						
5.01-5.50 mmol/L (90-99 mg/dL) (n = 3)	7.5 [135]	75.9	5.9	1.0	18.2	2.3
5.51-6.00 mmol/L (99-108 mg/dL) (n = 139)	8.1 [146]	73.6	4.1	0.9	22.3	2.6
6.01-6.50 mmol/L (108-117 mg/dL) (n = 48)	8.1 [146]	73.2	4.1	0.9	22.7	2.0
6.51-7.00 mmol/L (117-126 mg/dL) (n = 19)	8.6 [155]	70.4	2.3	0.4	27.4	2.5
7.01-7.50 mmol/L (126-135 mg/dL) (n = 7)	9.4 [169]	62.7	1.4	0.2	35.8	3.9

Personal glucose targets above 6.5 mmol/L (117 mg/dL) were associated with significantly less time in target range (3.9-10.0 mmol/L [70-180 mg/dL]; 6.5-7.0 mmol/L [117-126 mg/dL] bin: mean difference −3.2 percentage points [95% CI: −5.3 to −1.2; *P* < .001]; 7.0-7.5 mmol/L [126-135 mg/dL] bin: −10.8 percentage points [95% CI: −14.1 to −7.6; *P* < .001]) (Table 1), but the time in target only dropped below the recommended 70% when the personal target was set >7.0 mmol/L (126 mg/dL). The reduced time in target with higher personal targets occurred in parallel with significantly increased time above 10.0 mmol/L (180 mg/dL) (6.5-7.0 mmol/L [117-126 mg/dL] bin: mean difference 5.1 percentage points [95% CI: 3.0-7.2; *P* < .001]; 7.0-7.5 mmol/L [126-135 mg/dL] bin: 13.5 percentage points [95% CI: 10.2-16.9; *P* < .001]).

Lower personal targets were associated with increased time with glucose levels <3.9 mmol/L (70 mg/dL; 5.0-5.5 mmol/L [90-99 mg/dL] bin: mean difference −0.57 percentage points [95% CI: −1.00 to −0.14; *P* < .005]) although the data set was relatively small (Table 1). Only personal targets >6.5 mmol/L (117 mg/dL) were associated with significantly lower time <3.9 mmol/L (70 mg/dL) (Figure 1) (6.5-7.0 mmol/L [117-126 mg/dL] bin: −1.85 percentage points [95% CI: −2.37 to −1.34; *P* < .001]; 7.0-7.5 mmol/L [126-135 mg/dL] bin: −2.68 percentage points [95% CI: −3.49 to −1.86; *P* < .001]). No personalized targets were associated with significant differences in time with glucose levels <3.0 mmol/L (<54 mg/dL) (which was ≤1.0% across all bins).

There was no significant difference in glycemic outcomes with personal targets between 5.5 and 6.0 mmol/L (99-108 mg/dL) and between 6.0 and 6.5 mmol/L (108-117 mg/dL) suggesting that adjusting the personal target by >1 mmol/L (18 mg/dL) may be required for clinically meaningful changes to glucose outcomes.

Glucose metrics when different personal targets are applied are shown by study cohort in Table 2.

**Table 2.** Glycemic Metrics by Personal Glucose Target Bin Per Study Cohort.

	Metric	Study cohort				
		Very young children (1-7 years) (n = 73)	Children and adolescents (6-19 years) (n = 25)	Adolescents with new onset diabetes (10-17 years) (n = 44)	Adults (≥18 years) (n = 25)	Older adults (≥60 years) (n = 18)
Personal glucose target bin						
5.01-5.50 mmol/L (90-99 mg/dL)	Mean glucose (mmol/L) [mg/dL]	7.0 [126]	8.3 [149]	—	7.2 [130]	—
	Time 3.9-10.0 mmol/L (70-180 mg/dL)	79.3	68.3	—	80.2	—
	Time <3.9 mmol/L (70 mg/dL)	7.4	5.4	—	4.8	—
	Time <3.0 mmol/L (54 mg/dL)	1.1	1.1	—	0.8	—
	Time >10.0 mmol/L (180 mg/dL)	13.3	26.3	—	15.1	—
	Time >16.7 mmol/L (301 mg/dL)	1.1	4.2	—	1.5	—
5.51-6.00 mmol/L (99-108 mg/dL)	Mean glucose (mmol/L) [mg/dL]	7.9 [142]	8.5 [153]	8.4 [151]	7.8 [140]	7.4 [133]
	Time 3.9-10.0 mmol/L (70-180 mg/dL)	72.8	69.3	71.6	76.1	84.0
	Time <3.9 mmol/L (70 mg/dL)	5.7	3.9	3.1	3.7	2.2
	Time <3.0 mmol/L (54 mg/dL)	1.3	0.9	0.7	0.8	0.3
	Time >10.0 mmol/L (180 mg/dL)	21.6	26.8	25.4	20.2	13.8
	Time >16.7 mmol/L (301 mg/dL)	2.4	3.8	3.8	1.3	0.4
6.01-6.50 mmol/L (108-117 mg/dL)	Mean glucose (mmol/L) [mg/dL]	8.1 [146]	8.5 [153]	7.9 [142]	8.7 [157]	7.5 [135]
	Time 3.9-10.0 mmol/L (70-180 mg/dL)	72.2	70.8	77.4	70.2	84.8
	Time <3.9 mmol/L (70 mg/dL)	4.8	3.5	2.6	2.6	2.2
	Time <3.0 mmol/L (54 mg/dL)	1.1	0.8	0.5	0.7	0.3
	Time >10.0 mmol/L (180 mg/dL)	23.1	25.8	20.0	27.2	13.8
	Time >16.7 mmol/L (301 mg/dL)	1.9	3.3	2.0	2.9	0.4
6.51-7.00 mmol/L (117-126 mg/dL)	Mean glucose (mmol/L) [mg/dL]	8.8 [158]	9.9 [178]	8.4 [151]	—	7.5 [135]
	Time 3.9-10.0 mmol/L (70-180 mg/dL)	67.7	58.6	72.9	—	84.8
	Time <3.9 mmol/L (70 mg/dL)	2.6	1.7	1.8	—	1.7
	Time <3.0 mmol/L (54 mg/dL)	0.5	0.4	0.3	—	0.1
	Time >10.0 mmol/L (180 mg/dL)	29.6	39.7	25.4	—	13.5
	Time >16.7 mmol/L (301 mg/dL)	2.9	7.0	1.4	—	0.1
7.01-7.50 mmol/L (126-135 mg/dL)	Mean glucose (mmol/L) [mg/dL]	9.0 [162]	—	11.0 [198]	—	9.8 [176]
	Time 3.9-10.0 mmol/L (70-180 mg/dL)	66.0	—	49.5	—	59.5
	Time <3.9 mmol/L (70 mg/dL)	1.8	—	1.3	—	0.1
	Time <3.0 mmol/L (54 mg/dL)	0.3	—	0.2	—	0.0
	Time >10.0 mmol/L (180 mg/dL)	32.2	—	49.2	—	40.5
	Time >16.7 mmol/L (301 mg/dL)	2.3	—	14.3	—	1.4

## Discussion

Discrete study cohorts showed differences in glucose control when applying similar personal targets. The burden of hypoglycemia was greatest in very young children even when higher personal targets were used (Table 2), reflecting the challenges of diabetes management in this population.^1^

Older adults were able to achieve >80% time in range with the application of higher personal targets up to 7.0 mmol/L (126 mg/dL), a threshold at which there was only 1.1% of time in hypoglycemia (<3.9 mmol/L [70 mg/dL]), suggesting that higher personal targets may be beneficial in this population.

A similar trend of reduced time spent in the target glucose range when higher algorithm target glucose settings are applied has also been reported with the Omnipod (Insulet Corporation, Massachusetts, US) closed-loop system and in real-world data from the Medtronic 780G (Medtronic, Northridge, California, US) closed-loop systems.^6,7^ The Medtronic 780G settings that predicted the highest time in range were an active insulin time of two hours and the lowest glucose target of 5.6 mmol/L (100 mg/dL).

Strengths of our analysis include the use of data from participants across a wide range of age demographics and from multicenter, multinational clinical trials. Limitations include the retrospective analysis and that personal targets were averaged over the 24-hour period. Selection of personal targets was determined by the user, which may reflect individual differences in assessment of hypoglycemia and hyperglycemia risk. The largest cohort of participants were very young children who have the greatest hypoglycemia burden; however, this was accounted for in the analysis.

In conclusion, personal targets are a well-accepted and useful tool to individualize glucose control to suit the needs of the user. Our analysis allows users and healthcare professionals to understand the impact of personal target adjustments on glucose control and the recommended glycemic targets.^8^
